# Prevalence, Demographics, and Treatment Characteristics of Visual Impairment due to Diabetic Macular Edema in a Representative Canadian Cohort

**DOI:** 10.1155/2012/159167

**Published:** 2012-12-11

**Authors:** Robert J. Petrella, Julie Blouin, Brian Davies, Martin Barbeau

**Affiliations:** ^1^Aging, Rehabilitation and Geriatric Care Program, Lawson Health Research Institute, London, ON, Canada N6C 2R5; ^2^Individual Health Outcomes Inc., ON, Canada N5X 3W7; ^3^Parkwood Hospital, Aging, Rehabilitation and Geriatric Care Research Center, B-3002, 801 Commissioners Rd E., London, ON, Canada N6C 5J1; ^4^Outcomes Research, Novartis Pharmaceuticals Canada Inc., Dorval, QC, Canada H9S 1A9

## Abstract

Diabetic macular edema (DME) is the leading cause of blindness in the diabetic population. However, there is limited understanding of the epidemiology of DME with visual impairment (VI) and treatment in patients with diabetes in Canada. This observational, retrospective study used records from the Southwestern Ontario database to observe the demographics, prevalence, and treatment characteristics of VI due to DME compared to a healthy population in a real-world Canadian setting. Data was compared between a cohort of 8,368 diabetic (type 1 or 2) patients, who were ≥18 years old and had a diagnosis of DME with VI (visual acuity <20/40 in Snellen equivalent), and 76,077 age- and gender-matched subjects representing a healthy population. Among diabetic patients, prevalence of DME was 15.7%, and prevalence of VI due to DME was 2.56%. Laser monotherapy was the most frequently used treatment. Public funding covered costs for 85% of persons with DME while 18% were paid for with private funds. This study provides insight into the demographics, prevalence, and treatment of VI due to DME in a representative Canadian cohort. This data can help to inform evaluation of current DME treatment patterns and of proposed new treatment on drug plan budgets in Canada.

## 1. Introduction

Diabetic macular edema (DME), a complex disease of multifactorial origin, is the leading cause of blindness in the diabetic population [1]. Clinically significant macular edema, as defined by the Early Treatment Diabetic Retinopathy Study (ETDRS), includes any one of the following lesions: retinal thickening at or within 500 microns from the center of the macular; hard exudates at or within 500 microns from the center of the macular associated with thickening of the adjacent retina; an area or areas of retinal thickening at least 1 disk area in size, at least part of which is within 1 disk diameter of the center of the macular [2]. The common pathway that results in DME is disruption of the blood-retinal barrier [1]. As macular edema (ME) develops, blurring occurs in the middle or to the side of the central visual field. If untreated, ME can lead to vision loss. DME is closely associated with the duration and type of diabetes a patient has and the degree of diabetic retinopathy that is present [3]. 

The Wisconsin Epidemiologic Study of Diabetic Retinopathy (WESDR) reported the 25-year cumulative incidence of ME to be 29%, with annualized incidences of ME at 2.3%, 2.1%, 2.3%, and 0.9% in the first, second, third, and fourth follow-up periods of the study, respectively [4]. Findings from the Diabetes Control and Complications Trial (DCCT) study [5] reported that 27% of type 1 diabetic patients developed DME within 9 years of diabetes onset [1]. 

In a systematic review of epidemiology of diabetic retinopathy and macular edema (ME) Williams et al. (2004) found that a majority of the 359 articles included in the review were dominated by large studies like the WESDR, DCCT, ETDRS, and the United Kingdom Prospective Diabetes Study (UKPDS) [6]. Studies were highly heterogeneous in terms of subject selection with variable inclusion criteria relating not only to age, ethnicity, and comorbidity, but also to diabetic retinopathy status, assessment, and classification as well. Observing inconsistencies between epidemiological studies and conflicting reports of prevalence and incidence of diabetic retinopathy and ME in diabetic populations, the review stressed the importance of capturing and monitoring new epidemiological data to help to implement and assess effectiveness and impact of emerging therapies for diabetic retinopathy and its complications [6].

There is very limited understanding of the epidemiology and disease burden of DME and information on the current state of DME with visual impairment (VI) and treatment in patients with diabetes in Canada. This study examines the prevalence, demographics, and treatment characteristics of VI due to DME in a real-world Canadian setting. 

## 2. Methods

An observational, retrospective, noninterventional study was conducted using records from the Southwestern Ontario (SWO) database—a longitudinal population-based database of more than 170,000 patients in 53 family practice clinics in Southwestern Ontario, Canada.

The study cohort was identified using the following inclusion criteria:

- patients with diabetes (type 1 or type 2) and diagnosis of DME with visual impairment (defined as best corrected visual acuity <20/40 in Snellen equivalent) made by an ophthalmologist between January 1, 2008 and December 31, 2009, or the latest data available in the database with complete data for three months and after the index date (the date of the first diagnosis of DME during the study period until December 31, 2009);
- subjects of 18 years and older.

Exclusion criteria were as follows:

- patients without follow-up data to 1 year;
- patients diagnosed with age-related macular degeneration;
- patients diagnosed with a nondiabetes retinal vascular disease (i.e., retinal disease with a vascular origin including atherosclerosis or hypertension);
- patients who were participating in an ophthalmological clinical trial during the study period (if we have access to this information in the southwestern Ontario database).

Data included in this study comprised patient characteristics and demographics, prevalence, cardiovascular comorbidity and events, and medication coverage. Visual acuity was performed by a trained ophthalmologist or optometrist.

In order to compare characteristics and comorbidities of patients with DME to those of the general population, a control cohort was extracted by random selection of age- and gender-matched subjects >18 years by clinic location.

Initial extractions of control cohort, and the cohort of patients with diabetes and DME with VI, were accomplished utilizing International Classification of Disease codes (ICD9/ICD10), reviewing patient charts for text entries of symptoms that supported a diagnosis of diabetes and DME and concomitant comorbidity, and reviewing patient treatment records unique to DME with VI, including consultation notes and hospital discharge summaries. Demographic characteristics, comorbidities, and treatment were reported. In total, data from 8,368 patients with type 1 or 2 diabetes and from 76,077 people comprising the control cohort were extracted for this analysis. 

### 2.1. Description and Validity of the Southwestern Ontario (SWO) Database

The SWO database has recorded patient level data on the clinical diagnoses at each visit, symptoms corroborating the diagnoses, clinical data, prescribed treatments including lifestyle interventions and medications, physician visits, hospitalizations, and diagnostic/laboratory test results, allowing for the conduct of patient level analyses, since 2000. Data from the 53 practices participating in the SWO database cohort are routinely updated on a quarterly basis with immediate reconciliation at the point of care. The quarterly activity is triggered by chart entry and billed activity for patient encounters. All practices included in the SWO database are part of a family practice research network involved in various audit and clinical research activities. Practices have consented to centralized accrual of clinical data from the patient record (UWO IRB 09572). All records are anonymous and conform to current confidentiality industry standards. Each patient's Ontario Health Insurance Plan (OHIP) number is assigned a unique patient identification number in the SWO database. To protect the privacy of patient's medical information, a 128-bit SSL certificate is installed on the production SWO Web Server. The industry standard data protection method ensures the security of data during transmission across the Internet. Validation studies of the SWO database confirming the quality and completeness of the recorded data show good agreement between estimates of the prevalence of cardiovascular risk factors obtained from the SWO database and other published estimates [7–10]. Moreover, there is a correlation between the SWO database and national data (i.e., IMS, Brogan PharmaStat) on the utilization of prescription medication (personal communication, Petrella; Kamino, IMS). 

### 2.2. Statistical Analysis

All subjects meeting inclusion/exclusion criteria were analyzed to understand the demographics and treatment patterns of care. For treated patients, the treatment choices were characterized for each cell. The treatment pattern was related to clinical characteristics of patients, including type of coverage. 

For continuous variables, the mean, standard deviation, median, minimum, and maximum values were estimated. For categorical variables, the number and percentage of each category within an assessment were calculated for nonmissing data. 

## 3. Results 

### 3.1. Demographics

Please refer to Table 1 for demographic data for both the control and DME cohorts. One hundred sixteen (54%) patients with VI due to DME (defined as visual acuity <20/40) were male, 99 (44%) were female. The mean age of patients with VI due to DME was 64 years old, compared to 63 years old for patients with DME. The average age at diagnosis was 48 years old for patients with VI, compared to 52 years old for those with DME. The average duration of diabetes among patients with VI was 21 years compared to those with DME at 19 years.

A smaller percentage of patients with VI due to DME were Caucasian compared with control or those with DME (61%, *P* < .05). A larger proportion of aboriginals were affected by VI compared to the control or patients with DME (22%, *P* < .05). 

Please refer to Table 2 for disease characteristics data for both the control and DME cohorts. More patients with DME were overweight (BMI 25–29.5 kg/m^2^) (19%, *P* < .05), or obese (BMI ≥30 kg/m^2^) (12%, *P* < .05) than control. More patients with DME had hypertension (66%, *P* < .05), cardiovascular disease (Acute Coronary Syndrome, Myocardial Infarction, and Congestive Heart Failure) (25% overall, *P* < .05) than control. A higher percentage of patients with DME had a family history of diabetes (10%, *P* < .05), were smokers (21%, *P* < .05), and had a history of impaired fasting glucose (12%), impaired glucose tolerance (12%, *P* < .05), and higher blood glucose levels (7.3 ± 1.3 mmol/L).

### 3.2. Prevalence of Visual Impairment due to DME

Please refer to Table 3 for incidence and prevalence data for the DME cohort. Having DME is not static as per consultant notes: visual impairment, recorded from consult note abstraction, could include several episodes per patient during the observation period. Among diabetic patients, prevalence of DME was 15.7%. Most patients with DME were type 2 diabetic (83%). The prevalence of VI due to DME was 2.56% (annual incidence 0.37%). 

Please refer to Table 4 for data on visual impairment in the control and DME cohorts. Patients with VI on average had the disease for a longer duration and had a higher mean blood glucose level (Hb1AC = 7.6 ± 2.2) than those without VI. In patients with VI due to DME, 53% were classified as having focal, and 47% diffuse edema. 

### 3.3. Treatment Characteristics of Visual Impairment

Please refer to Table 5 for treatment characteristics data for patients with VI due to DME. One hundred fourteen (53%) patients with VI were identified as having focal, and 101 (47%) with diffuse edemas.The average time to first treatment was 27 ± 18 days. The mean lag time to second occurrence (relapse) was 59 ± 96 days. For both focal and diffuse edema, the most common treatment was laser monotherapy, used in 69% and 53% of cases, respectively. In patients with focal edema, Anti-VEGF was used in 8%, and intravitreal triamcinolone acetonide (IVTA) in 3% of laser combo treatments. In patients with diffuse edema, anti-VEGF was used in 12%, and IVTA in 15% of laser combo treatments. anti-VEGF alone was used in treatment of 18% of patients with focal edema, and 15% of patients with diffuse edema. Meanwhile, IVTA was used to in 1% of patients with focal edema, and 3% of patients with diffuse edema. 

### 3.4. Paying for Treatment

Public funding covered costs for 85% of persons with DME, 18% were paid for with private funds, and 1% from out-of-pocket resources.

## 4. Discussion

DME is a chronic disease. The natural progression of DME leads to a significant vision loss within 2 years in half of individuals [11]. Epidemiological data on visual impairment due to DME are sparse and vary widely across countries. This was the first Canadian study to assess the incidence and prevalence of visual impairment due to DME.

This study has some limitations. DME can be associated with severity of hypertension alone. Given that blood pressure levels and % of patients with hypertension were similar between those with and without DME, we do not believe that hypertension would contribute differently to outcomes in those with DME although this remains a possibility. Treatment timelines were taken from chart records and were not verified with patients, and adherence with treatment could not be captured. Furthermore, the validity of the diagnostic algorithms relies on the premise that the coding ophthalmologist accurately diagnosed the presence or absence of DME as well as the classification and treatment of focal and diffuse types of edema in patients with DME. Although classification of macular edema into focal and diffuse types is a common practice, a critical review by Browning et al. (2008) found little evidence that characteristics of macular edema described by the terms focal and diffuse are of clinical significance in that they do not adequately help to explain variation in visual acuity or response to treatment [12]. Finally, difficulties in interpreting data contained in consultation notes, as well as data concerning number of episodes requiring consultation and treatment, may have resulted in some inconsistencies in data capture.

Overall, however, unlike pharmaceutical administrative databases which use service diagnosis codes to correlate with prescription use, this database allows for a linkage to be made between prescribed medications and disease correlates. As well, previously conducted validation studies of the SWO database confirming the quality and completeness of the recorded data show good agreement between estimates of the prevalence of cardiovascular risk factors obtained from the SWO database and other published estimates [7–10]. Moreover, there is a correlation between the SWO database and national data (i.e., IMS, Brogan PharmaStat) on the utilization of prescription medication (personal communication, Petrella; Kamino, IMS). 

## 5. Conclusions

This study in a real-world setting among patients with diabetes provides insight into the prevalence, demographics, and treatment characteristics of visual impairment due to DME in a representative Canadian cohort. As such, it can help to inform evaluation of the current DME treatment patterns and help to populate budget impact models evaluating the impact of introducing new treatment on drug plan budgets in Canada. 

## Figures and Tables

**Table 1:** Demographics of the populations investigated.

	Control cohort (*N* = 76,077)	Patients with DME (*N* = 1,316)	Patients with DME (<20/40) (*N* = 215)
Average age (years)	69	63	64
Average duration of diabetes (years)	n/a	19	21
Average age at diagnosis of DME (years)	n/a	52	48
Age distribution [*n* (%)]			
Males <40	14,455 (19%)	92* (7%)	6 (3%)
Males 40–59	1,932 (17%)	236* (18%)	26 (12%)
Males 60–69	3,803 (5%)	263* (20%)	67 (31%)
Males 70+	6,086 (8%)	92 (7%)	11 (5%)
Females <40	14,454 (19%)	158* (12%)	24 (11%)
Females 40–59	11,411 (15%)	184 (14%)	38 (18%)
Females 60–69	3,804 (5%)	234* (8%)	34 (16%)
Females 70+	9,129 (12%)	53* (4%)	9 (5%)
Sex [*n* (%)]			
Male	37,551 (49%)	671 (51%)	116 (54%)
Female	38,526 (51%)	645 (49%)	99 (44%)
Ethnicity [*n* (%)]			
Caucasian	59,340 (76%)	934 (71%)	131** (61%)
Aboriginal	6,847 (9%)	158 (12%)	47** (22%)
Hispanic	3,043 (4%)	92* (7%)	0
South Asian	3,055 (5%)	79 (6%)	37 (17%)
Asian	1,521 (2%)	39 (3%)	0
African descent	1,506 (2%)	13 (1%)	0

*Significantly different from cohort. 1-tail test. *P* < .05.

**Significantly different from DME cohort. 1-tail test. *P* < .05.

**Table 2:** Disease characteristics of the populations investigated.

	Control cohort (*N* = 76,077)	Patients with type 2 diabetes on oral meds (*N* = 6,086)	Patients with type 1 diabetes (*N* = 2,282)	Patients with DME (*N* = 1,316)
Overweight (BMI = 25–29.9 kg/m^2^) [*n* (%)]	3,799 (5%)	1,095* (18%)	208 (9%)	250* (19%)
Obesity (BMI ≥30 kg/m^2^) [*n* (%)]	1,522 (2%)	791* (13%)	92 (4%)	158* (12%)
BMI, male (mean ± SD)	25 ± 13.9	29 ± 14.0	24 ± 9	29 ± 9
BMI, female (mean ± SD)	24 ± 14.1	28 ± 14.7	26 ± 11	30 ± 7
Family history, type 2 diabetes [*n* (%)]	782 (1%)	487* (8%)	274* (2%)	132* (10%)
Smoking [*n* (%)]	3,804 (5%)	1,339* (22%)	416* (18%)	276* (21%)
Hypertension [*n* (%)]	10,650 (14%)	4,138* (68%)	1,529* (67%)	868* (66%)
Systolic blood pressure (mean ± SD)	131 ± 15.4	133 ± 15.3	133 ± 14	136 ± 11
Diastolic blood pressure (mean ± SD)	77 ± 8.5	76 ± 8.5	81 ± 3	82 ± 9
Dyslipidemia [*n* (%)]	7,603 (10%)	2,921* (48%)	251 (11%)	166* (12%)
History of impaired fasting glucose (IFG) [*n* (%)]	0 (0%)	791* (13%)	16 (1%)	154* (12%)
History of impaired glucose tolerance (IGT) [*n* (%)]	780 (1%)	1,095* (18%)	11 (1%)	160* (12%)
Hb1Ac (mean ± SD)	5.1 ± 0.6	6.7 ± 1.8	6.5 ± 1.1	7.3 ± 1.3
History of vascular disease (coronary, cerebrovascular, or peripheral) [*n* (%)]	9,129 (12%)	1,643* (27%)	319 (14%)	368* (28%)
Acute coronary syndrome (ACS) [*n*, (%)]	4,567 (6%)	1,947* (32%)	479* (21%)	276* (21%)
Acute				
Myocardial infarction (MI) [*n* (%)]	799 (1%)	487* (8%)	387* (17%)	237* (18%)
Stroke [*n* (%)]	714 (1%)	182 (3%)	12 (1%)	91* (7%)
Percutaneous coronary transluminal arthroplasty (PCTA) [*n* (%)]	689 (1%)	61 (1%)	25 (1%)	27 (2%)
Coronary artery bypass graft (CABG) [*n* (%)]	701 (1%)	65 (1%)	32 (1%)	13 (1%)
Peripheral arterial disease (PAD) [*n* (%)]	215 (<1%)	121 (2%)	10 (1%)	66* (5%)
Congestive heart failure (CHF) [*n* (%)]	755 (1%)	426* (7%)	46 (2%)	105* (8%)
History gestational diabetes (females) [*n* (%)]	181 (<1%)	60 (1%)	50 (2%)	26 (2%)
Chronic kidney disease [*n* (%)]				
Stage 1	0 (0%)	88 (1%)	12 (1%)	29 (2%)
Stage 2	0 (0%)	92 (1%)	68 (3%)	55 (4%)
Stage 3	737 (1%)	118 (2%)	161 (7%)	79 (6%)
Stage 4	2,282 (3%)	212 (4%)	114 (5%)	75 (6%)
Stage 5 (end stage renal disease)	766 (1%)	109 (2%)	42 (2%)	37 (3%)
Dialysis	(<1%)	175 (3%)	37 (2%)	39 (3%)
Microvascular complications [*n* (%)]				
Amputations	281 (1%)	177 (3%)	35 (2%)	28 (2%)
Peripheral neuropathy	741 (2%)	365 (6%)	17 (1%)	77 (6%)
Nephropathy	1,521 (2%)	28 (4%)	71 (3%)	94 (7%)

*Significantly different from control. 1-tail test. *P* < .05.

**Table 3:** Incidence and prevalence.

	Patients with DME (*N* = 1,316)	Patients with DME (<20/40) (*N* = 215)
Visual impairment	**829 (63%) **	**—**
Prevalence	15.7%	**2.56% **
Incidence	—	**0.37% **

Visual impairment (VI) is defined as visual acuity <20/40.

**Table 4:** Visual impairment in patients with DME.

	Control (*N* = 76,077)	Patients with DME (*N* = 1,316)		Patients with DME (<20/40) (*N* = 215)	
Age (years)	69	63		64	
HbA1c at diagnosis (mean ± SD)	6.3 ± 0.3	7.4 ± 1.7		7.6 ± 2.2	
Right eye affected [*n* (%)]	n/a	486 (37%)		96 (45%)	
Left eye affected [*n* (%)]	n/a	632 (48%)		92 (43%)	
Both eyes affected [*n* (%)]	n/a	197 (15%)		26 (12%)	

Type*	n/a	Focal	Diffuse	Focal	Diffuse
		750 (57%)	566 (43%)	114 (53%)	101 (47%)

*Diffuse edema is associated with a paucity of lipid exudates. Focal edema is associated with the presence of lipid and lipid rings [1].

**Table 5:** Treatment characteristics of patients with VI due to DME.

	Patients with VI due to DME (*N* = 215)	
Average time to treatment (days) (mean ± SD)	27 ± 18	
Lag to second treatment (days) (mean ± SD)	59 ± 96	

Type	Focal	Diffuse

Number of patients [*n* (%)]	114 (53%)	101 (47%)

Treatment type (% of all treatments recorded in patients' charts)*		

Anti-VEGF monotherapy	18.2%	15.0%
Intravitreal Triamcinolone Acetonide (IVTA)	1.0%	3.0%
Other	0.5%	1.9%
Laser mono	69.3%	53.3%
Laser combo		
Laser—anti-VEGF	7.8%	12.1%
Laser—IVTA	3.1%	15.0%

*Some patients received more than one type of treatment; anti-VEGF: anti-vascular endothelial growth factor.
